# Different Short-Term Mild Exercise Modalities Lead to Differential Effects on Body Composition in Healthy Prepubertal Male Rats

**DOI:** 10.1155/2015/404201

**Published:** 2015-01-28

**Authors:** D. M. Sontam, M. H. Vickers, J. M. O'Sullivan, M. Watson, E. C. Firth

**Affiliations:** ^1^The Liggins Institute, University of Auckland, Auckland 1142, New Zealand; ^2^Gravida: National Centre for Growth and Development, University of Auckland, Auckland 1142, New Zealand; ^3^Department of Medicine, University of Auckland, Auckland 1142, New Zealand; ^4^Department of Sports and Exercise Science, University of Auckland, Private Bag 92019, Auckland 1142, New Zealand

## Abstract

Physical activity has a vital role in regulating and improving bone strength. Responsiveness of bone mass to exercise is age dependent with the prepubertal period suggested to be the most effective stage for interventions. There is a paucity of data on the effects of exercise on bone architecture and body composition when studied within the prepubertal period. We examined the effect of two forms of low-impact exercise on prepubertal changes in body composition and bone architecture. Weanling male rats were assigned to control (CON), bipedal stance (BPS), or wheel exercise (WEX) groups for 15 days until the onset of puberty. Distance travelled via WEX was recorded, food intake measured, and body composition quantified. Trabecular and cortical microarchitecture of the femur were determined by microcomputed tomography. WEX led to a higher lean mass and reduced fat mass compared to CON. WEX animals had greater femoral cortical cross-sectional thickness and closed porosity compared to CON. The different exercise modalities had no effect on body weight or food intake, but WEX significantly altered body composition and femoral microarchitecture. These data suggest that short-term mild voluntary exercise in normal prepubertal rats can alter body composition dependent upon the exercise modality.

## 1. Introduction

Bone is sensitive to the mechanical stimuli imposed on it and responds to the loads applied largely by changing its mass and morphology [1, 2]. Physical activity thus has a vital role in regulating and improving bone strength. The strength of the response is age dependent with the prepubertal period being the most effective stage for exercise to have maximal effect on bone mass [3–7].

Evidence is accumulating that supports the hypothesis that changes in bone mass and architecture as a result of exercise at a young age are retained even after the cessation of exercise. For instance, in a school-based impact exercise intervention program that spanned seven months and involved children who were 10.2 ± 0.6 years old, the bone mineral content (BMC) in the proximal femur of the group which participated in the exercise program was 3.6% higher than in the control group. Of note, the BMC of the intervention group remained 1.4% higher than that of the control group eight years after the cessation of the exercise program [8]. Likewise, bone health parameters in 75-year-old Swedish men were significantly positively correlated with the amount of competitive sport they participated in between the ages of 10 and 35 years [9]. Similar observations have been made in studies of bone health in gymnasts [5], tennis players [10], and weight lifters [11, 12]. Thus exercise not only has immediate positive effects on improving bone health in the young (reduction in child fracture risk) [13] but also appears to have the potential to mitigate bone loss in later life.

Consistent with studies in humans, work in animal models has shown that the effects of physical activity on bone are retained after exercise cessation. Four-week-old male Wistar rats that underwent treadmill training for 10 weeks had greater BMC and longer bones than the control group. The bone mass accrued through training was retained after 10 weeks of exercise cessation (detraining) [14]. Five-week-old Sprague-Dawley rats that were exercised three days per week for seven weeks had improved bone quantity and strength; these changes were still present when the rats were examined at two years of age [15]. However, other studies have shown that exercise-induced changes in bone are lost with detraining [16, 17]. Four-week-old female Sprague-Dawley rats that exercised on a treadmill for eight and twelve weeks had greater femoral wet weight and bone volume than the control groups. However, eight weeks of exercise followed by four weeks of cessation resulted in a decrease of bone mass to levels seen in sedentary controls [16]. In five-week-old male Sprague-Dawley rats, 14 weeks of treadmill running improved the size, mass, and strength of the femoral neck. These changes were partially retained during a subsequent deconditioning period of 14 weeks. But the changes eventually disappeared after 42 weeks of detraining [17]. The apparent contradictions in the results of these studies [14–17] may be due to one or more factors which include age and gender of the animals, exercise modality and duration, skeletal site examined, and experimental design [15, 17].

There is a gap in our knowledge as to the ability of mild exercise to influence bone development when the exercise begins and ends before puberty is reached. For example, although the studies mentioned above [14–17] involved animals that may have been prepubertal when the studies commenced, the duration of the exercise protocols meant that the animals were well past puberty by the end of the experimental period. Therefore, to explore the effect of prepubertal exercise on bone development, we conducted a controlled study where 21-day-old male Sprague-Dawley rats were allowed to exercise voluntarily (i.e., wheel running or rising to a bipedal stance) for a period of 15 days spanning the period between weaning and puberty.

## 2. Materials and Methods

### 2.1. Study Design

Time-mated female Sprague-Dawley rats were used to generate 24 offspring derived from a total of four litters. Litter size was limited to eight pups per dam to standardize nutrition until weaning. On D_20_, preexercise body composition was quantified, while under light isoflurane anaesthesia, using dual energy X-ray absorptiometry with dedicated small animal software (DXA, Lunar Hologic, GE, Waltham, MA, USA). Rats were allocated to one of three treatment groups ensuring no statistically significant differences with respect to starting body weight and total fat percentage and controlling for litter of origin. The exercise groups consisted of control (CON), bipedal stance (BPS), and wheel exercise (WEX) groups. All animals were housed in a temperature (19.9°–21.8°C) and light-dark cycle (12 : 12, lights on at 6 a.m.) controlled facility with* ad libitum* access to food and water (Diet 2018, Teklad Global 18% Protein Rodent Diet, Harlan Teklad, USA). Rats were housed in pairs except for two intervals (on D_27–29_ and D_33–35_) during which the BPS and WEX rats underwent individual exercise monitoring as detailed below. The experimental period lasted from D_21_ to D_35_ and the animals were culled on D_36_.

Water and food intake and body weight were monitored regularly throughout the study in order to examine possible effects of exercise on caloric intake. The amount of food consumed was determined as the difference between the residual and initial feed weights. Caloric intake, defined as the number of kilocalories (Kcal) consumed per gram of body weight, was calculated for each pair of rats using feed consumption information and the caloric density of the diet (3.1 kcal/g).

The CON group was housed in pairs in standard cages. The BPS group was housed in pairs in a specially modified BPS cage [6, 18]. There was an initial three-day exercise familiarization period (D_21–23_) during which the feed height was gradually raised from a standard height of 90 mm to its full height of 220 mm. Raising the feed required the rats to fully extend the tibiotarsal and femorotibial joints. During the individual exercise period which lasted for 44 hours on D_27–29_ and 36 hours on D_33–35_, the two rats in each cage were separated by a clear plastic “buddy barrier” in order to minimize stress due to cage-mate separation.

WEX rats were pair-housed in cages containing an activity wheel (Model 80859, Lafayette Instrument, Lafayette, IN, USA) connected to a wheel control and counter (Model 86070A) which was used to monitor the exercise levels in one-hour intervals throughout the experimental period. Data were recorded using dedicated monitoring software (Model 86065). During the first day of the familiarization period (D_21_), the wheel was locked and the animals were allowed to explore their new environment. The wheel was unlocked on D_22_ and resistance of the wheel set to zero and remained so for the rest of the trial period. During the individual exercise periods (47 hours on D_27–29_ and 36 hours on D_33–35_), each rat was in its own wheel-instrumented cage. Cages were made of clear plastic, and the cage-mates were placed immediately adjacent to each other, so that the rats could see each other during separation to minimize the stress response due to cage-mate separation. All animal work was approved by the University of Auckland Animal Ethics Committee.

### 2.2. Whole Body Composition Analyses

DXA was performed on all animals on D_20_ and D_35_ to determine whole body composition (lean and fat mass), fat to lean mass ratio, bone mineral content (BMC), and areal bone mineral density (_a_BMD). D_20_ DXA measurements were used to allocate the rats to exercise groups.

### 2.3. Tissue Collection

All animals were culled on D_36_ by decapitation under isoflurane anaesthesia following an overnight fast. Fasting blood glucose measurements were made on tail blood samples using a glucose meter (Precision Xtra, Abbott, USA). Trunk blood was collected into heparinized tubes stored on ice, centrifuged and the plasma was separated and stored at −20°C until analysis. Epididymal and retroperitoneal fat pads were removed, weighed, snap-frozen in liquid nitrogen and stored at −80°C. The left femur was dissected and sectioned into proximal, mid, and distal sections, snap-frozen in liquid nitrogen, and stored at −80°C. The right femur was stored in 70% ethanol and was used for *μ*CT analysis.

### 2.4. Plasma and Fecal Analysis

Fasting plasma samples were analyzed for insulin and leptin using rat-specific commercial ELISAs (90060 and 90040, resp., Crystal Chem, Downers Grove, IL, USA) according to the manufacturers' instructions. Plasma corticosterone and testosterone concentrations were measured using LC MS/MS as previously described [19]. In addition to plasma corticosterone, which exhibits a diurnal variation, fecal samples were also collected and corticosterone measured via mass spectrometry to reflect a more stable time course as per our previous publications [18].

### 2.5. Microcomputed Tomography (*μ*CT)

The right femur of each animal was cleaned to remove all soft tissue and placed in 70% ethanol at 4°C. Femur length was measured using sliding calipers. Femurs were scanned using SkyScan 1172 *μ*CT scanner (Bruker, Aartselaar, Belgium) as previously described [20], with X-ray voltage 65 kV, 1 mm aluminium filter, and isotropic voxel size of 8 *μ*m. After standardized reconstruction using SkyScan NRecon software, the datasets were analyzed using SkyScan CT-analyzer software (CTAn, SkyScan, Aartselaar, Belgium). Volumes of interest (VOI) were selected and analyzed in two regions of the distal femur: for trabecular imaging, the volume of interest (VOI) was 2.32 mm proximal to the distal femoral physis and extended 2 mm in the proximal direction; the cortical bone-imaging site was 5.6 mm proximal to the physis and extended 0.8 mm proximally.

### 2.6. Statistical Analyses

All statistical analyses were carried out using SigmaPlot (V12.5, SysStat Software Inc., Ca, USA) and GraphPad Prism 6.0 (GraphPad Software, San Diego, CA, USA). One-way analysis of variance (ANOVA) was employed to determine significant differences between the three treatment groups. The alpha value was set at 0.05. If the ANOVA showed a significant difference (*P* value <0.05), multiple comparison testing was performed using the Holm-Sidak procedure. Differences in caloric intake between groups over the trial period were tested by mixed ANOVA using IBM SPSS Statistics 21 (release 21.0.0) with an alpha level of 0.05. Body weight data were also analysed using repeated measures ANOVA.

## 3. Results

### 3.1. Animals: Weight Gain and Food Intake

**Figure 1:**
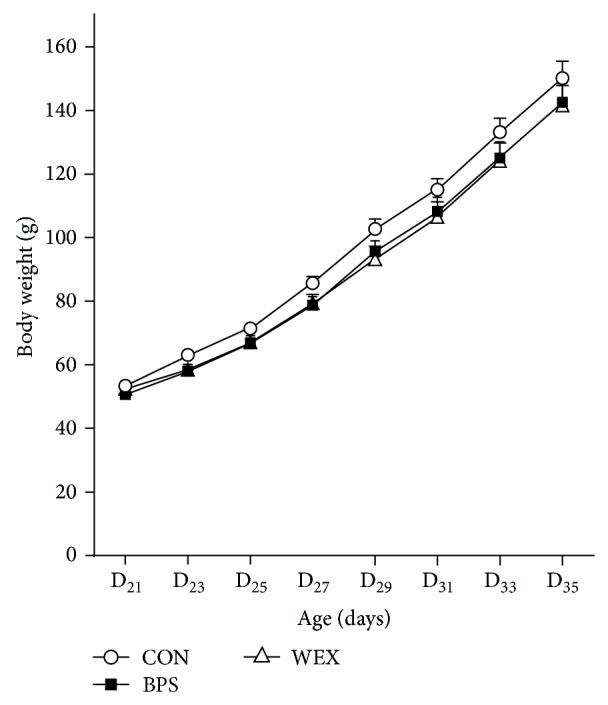
Body weights of prepubertal rats in control, BPS, and WEX exercise groups. CON: control; BPS: bipedal stance; WEX: wheel exercise. Data are presented as mean ± SEM, *n* = 8 per group.

**Figure 2:**
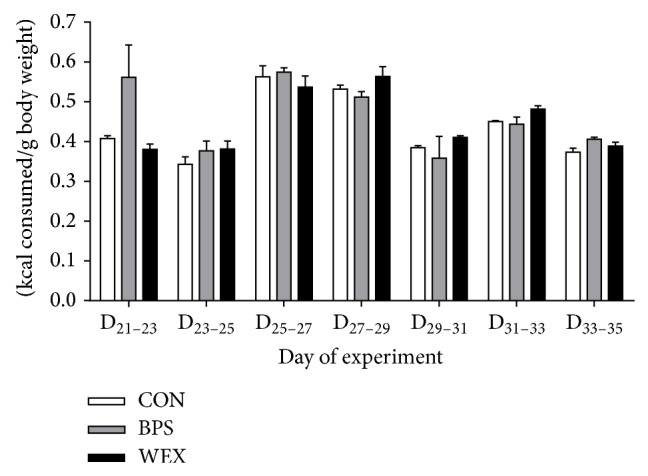
Caloric intake of rats during the prepubertal exercise period. Caloric intake was obtained by dividing the number of Kcal consumed by the body weight of the animals. All data are on a per cage basis and presented as mean ± SEM. CON: control; BPS: bipedal stance; WEX: wheel exercise.

The group mean weights of the animals on D_21_ were not significantly different (CON: 53.44 ± 1.18 g; BPS: 50.68 ± 1.49; WEX: 52.49 ± 3.88). The trajectory of weight gain over the course of the experimental period was similar between the groups (Figure 1) and the final body weights of the groups were CON: 150.20 ± 5.35 g; BPS: 142.65 ± 5.92; WEX: 141.61 ± 6.29. The total weight gain, defined as the difference between the final and initial body weights, was highest in CON (96.77 ± 4.52 g) followed by BPS (91.98 ± 4.71) and then WEX (89.12 ± 5.6). Neither final body weight nor the total weight gain was significantly different between the groups. There were no significant differences in caloric intake between the groups across the treatment period. Although the BPS group appeared to have a high caloric intake between D_21_ and D_23_ (Figure 2) this difference did not reach statistical significance.

### 3.2. Exercise

**Figure 3:**
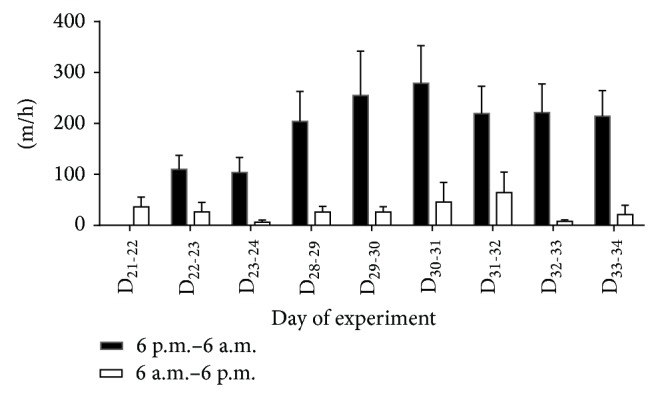
Comparison of wheel exercise (WEX) activity during the light and dark periods. For D_21–24_ and D_30–32_, data represent the mean exercise for pairs of rats (*n* = 4 pairs). For each of D_28-29_, D_29-30_, D_32-33_, and D_33-34_, data are means measurements for individual rats (*n* = 8). Data are presented as mean ± SEM.

The BPS and WEX animals exercised without any observable adverse health effects. The propensity for each animal to exercise varied among the animals. The total wheel exercise distance on the first day the wheel was unlocked was 1801 ± 493 m day^−1^ cage^−1^ (i.e., sum of distance for two rats, which were housed in pairs at this stage). WEX activity depended on the time of day and was considerably lower during the light than the dark period throughout the 15-day trial (Figure 3). For instance, on D_22-23_, the mean distance ran by the four pairs of WEX animals was 1453 ± 474 m during the dark period but only 348 ± 104 m between 6 a.m. and 6 p.m. in the succeeding light period on D_23_.

The exercise records of the two periods in which the wheel exercise of each individual rat was quantified showed that the amount of exercise varied greatly between individual rats. For instance, the distances run ranged from 881 m day^−1^ to 6373 m day^−1^ during the first individual exercise period and from 916 m day^−1^ to 7376 m day^−1^ during the second individual exercise period.

### 3.3. Body Composition

**Figure 4:**
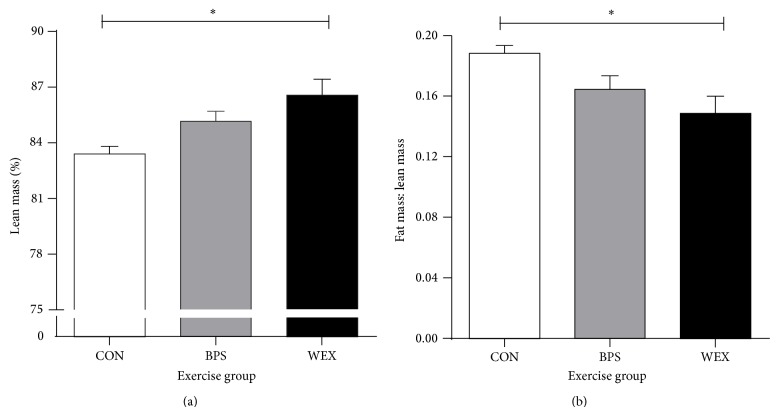
Comparison of body composition at D_36_ between the exercise groups. (a) The lean mass percentage of WEX was significantly higher than CON. (b) The fat mass to lean mass ratio was significantly lower in WEX compared to CON. CON: control; BPS: bipedal stance; WEX: wheel exercise. Data are presented as mean ± SEM; *n* = 8 per group. ^*^ *P* < 0.05 for CON versus WEX.

At the end of the exercise period (D_35_) the WEX group had small but significantly increased lean mass percentage than the CON and BPS groups (CON: 83.41 ± 0.4%; BPS: 85.16 ± 0.55; WEX: 86.56 ± 0.86, Figure 4(a)). There were no significant differences in percentage total fat mass as quantified by DEXA (15.51 ± 0.33%, 14.46 ± 0.72 and 13.33 ± 0.91 for CON, BPS, and WEX resp.). The fat/lean mass ratio was lower in WEX than CON and BPS groups (CON: 0.19 ± 0.01; BPS: 0.16 ± 0.01; WEX: 0.15 ± 0.01, Figure 4(b)).

**Figure 5:**
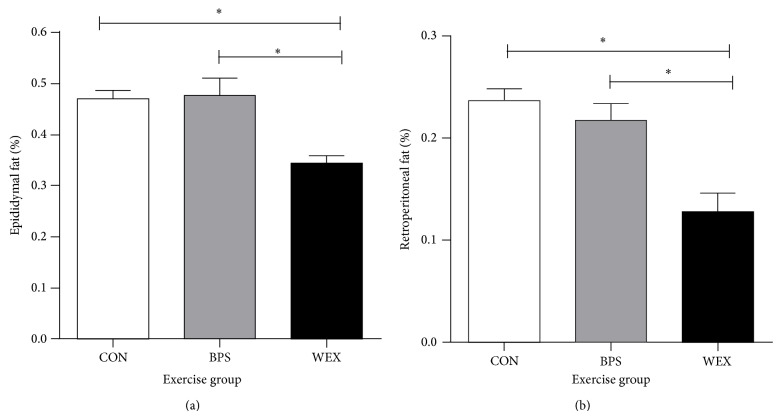
Epididymal (a) and retroperitoneal (b) fat weights of prepubertally exercised rats. WEX rats had a significantly lower percentage of epididymal fat and lower percentage of retroperitoneal fat. CON: control; BPS: bipedal stance; WEX: wheel exercise. Data are mean ± SEM; *n* = 8 per group. ^*^ *P* < 0.05.

Absolute weights of epididymal fat pads were significantly decreased in the WEX group compared to CON and BPS groups (CON (mg): 660 ± 35; BPS: 677 ± 57; WEX: 450 ± 23, *P* < 0.05 for CON and BPS versus WEX). When adjusted for body weight, the relative epididymal fat pad weights remained significantly decreased in the WEX group compared to CON and BPS groups (CON: 0.47 ± 0.02; BPS: 0.48 ± 0.03; WEX: 0.34 ± 0.01, Figure 5(a)). Similarly, absolute weights of retroperitoneal fat pads were significantly decreased in the WEX group compared to CON and BPS groups (CON (mg): 331 ± 18; BPS: 310 ± 32; WEX: 171 ± 27, *P* < 0.05 for CON and BPS versus WEX). When adjusted for body weight, the relative retroperitoneal fat pad weights remained significantly decreased in the WEX group compared to both CON and BPS groups (CON (%BW): 0.24 ± 0.03; BPS: 0.22 ± 0.02; WEX: 0.13 ± 0.021, Figure 5(b)).

### 3.4. Plasma and Fecal Analysis

Plasma concentrations of leptin (CON: 0.90 ± 0.14 ng/mL; BPS: 0.88 ± 0.06; WEX: 0.89 ± 0.11), insulin (CON: 0.17 ng/mL ± 0.02; BPS: 0.19 ± 0.03; WEX: 0.19 ± 0.03), and corticosterone (CON: 270.4 ± 66.1 ng/mL; BPS: 390.2 ± 53.4; WEX: 326.7 ± 88) were not significantly different between groups. Fecal corticosterone was not detectable in any of the samples analyzed. Blood glucose concentrations (mmol/L) were also similar between the groups (CON: 3.85 ± 0.17; BPS: 4.25 ± 0.17; WEX: 4.14 ± 0.21). Testosterone concentrations were low in all the animals confirming that these animals were prepubertal (CON: 71.9 ± 33.0 pg/mL; BPS: 65.0 ± 21.6; WEX: 67.3 ± 20.4).

### 3.5. Bone Parameters

**Figure 6:**
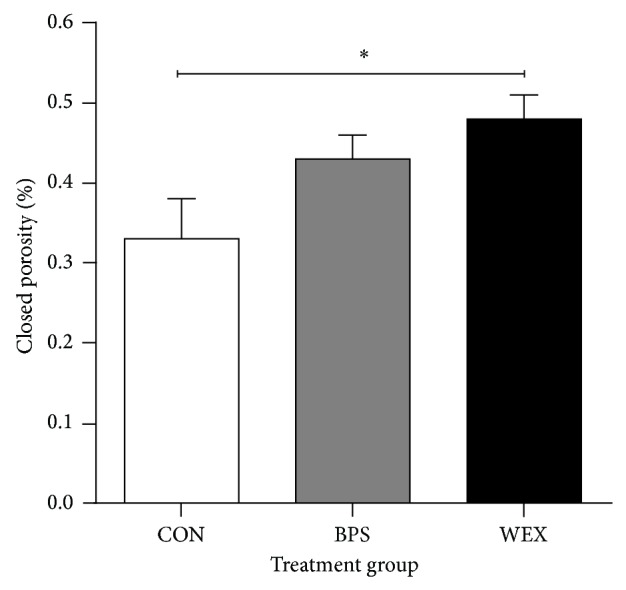
Closed porosity (%) in bones from prepubertally exercised rats. Percent closed porosity was significantly increased in WEX versus CON animals. CON: control; BPS: bipedal stance; WEX: wheel exercise. Data are mean ± SEM; *n* = 8 per group. ^*^ *P* < 0.05.

The trabecular and cortical parameters of the right femur are summarized in Table 1 and Figure 6. The three exercise groups had similar femur lengths (CON: 24.17 ± 0.2 mm; BPS: 23.80 ± 0.24 mm; WEX: 23.74 ± 0.35 mm). There were no significant between-group differences in the BMC (standardized to body weight) as measured by DXA. Cortical tissue mineral density (TMD) and trabecular bone mineral density (BMD) also showed no between-group differences. Cortical cross-sectional thickness was significantly greater in the WEX than the CON group. Numerically, cortical cross-sectional thickness was 15% higher in the WEX group, similar to the 12% higher cortical BMC in the WEX than CON group when values were corrected to body weight. Open and closed porosity was defined using the SkyScan software as follows: closed pore; 3D connected assemblage of space (black) voxels that is fully surrounded on all sides in 3D by solid (white) voxels. Conversely an open pore was defined as any space located within a solid object or between solid objects which has any connection in 3D to the space outside the object(s). Percent of closed porosity was higher in the WEX than in the CON group (Figure 6). The differences between CON and WEX with respect to percent of open porosity and total porosity approached statistical significance (*P* = 0.062 and *P* = 0.067 for open and total porosity, resp.).

## 4. Discussion

The onset of puberty does not occur at a fixed age in rats. In male rats, the first spermatozoa appear in seminiferous tubules by 40–45 days [21] and the first significant increase in plasma testosterone has been reported to occur between 40 and 50 days of age [22]. Evidence of balanopreputial separation, which occurs at the time of pubertal rise in androgen levels, is often used as an indicator of pubertal onset and this is known to occur at approximately 39 days of age in healthy male rats [23]. This evidence, taken together with the low plasma testosterone concentrations observed in rats in the current study serves to validate the prepubertal status of the animals.

Despite their young age, the animals were able and willing to exercise throughout the trial period. Importantly, the low plasma corticosterone concentration observed, which was not different between the groups, indicated that the exercised animals did not experience additional stress due to the exercise regime. Moreover, the WEX rats rapidly adapted to the exercise environment and immediately showed a clear circadian rhythm in their activity, running considerably greater distances during the dark period, in keeping with their nocturnal nature [24]. This exercise pattern persisted throughout the trial period, consistent with previous observations [25].

The amount of activity varied widely between the WEX rats as evidenced by the individual exercise records. For both periods in which exercise of each WEX individual was quantified, the individual which ran the greatest distance ran 7-8 times more than the individual that ran the least. Such differences in running activity are not uncommon even in rats of the same strain [26–28] and have been attributed to genetic, hormonal, physiological, and psychological factors [29].

Effects of voluntary exercise on body weight in rats are known to be highly variable [25, 26] and have been attributed to gender, strain, age, or experimental conditions [25]. Introduction of exercise (BPS or WEX) in the current study did not affect either the trajectory of weight gain during the trial period or the final body weight of the animals. Similar findings were reported elsewhere in studies in which young male rats (21 days–30 days old) voluntarily exercised on a wheel [25, 30, 31].

Although the absolute calorie consumption increased in all the groups as the experiment progressed, calorie consumption per gram of body weight remained similar over the course of the experiment. The fact that BPS animals' calorie intake was similar to the other groups indicates that the animals had little difficulty reaching the elevated food source. A lack of significant changes in food intake across the different exercise modalities confirms that the effects observed on reduced fat pad weight in the WEX group is resultant from the exercise itself and not a reduction in caloric intake.

Although the mean body weights of the groups did not show significant differences, the body composition of the WEX group differed significantly from that of the CON and BPS rats. WEX rats had a significantly higher percent lean mass and lower fat to lean mass ratio than the CON and BPS groups. The WEX group also had a significantly decreased absolute and relative epididymal and retroperitoneal fat mass compared to CON and BPS animals. Despite the changes in fat mass, plasma leptin concentrations, which have previously been shown to correlate positively to adipose tissue mass in both humans and rodents [32], were not significantly different between the exercise groups. This could relate to the young age of the animals and leptin-independent changes in body composition in rodents as reported by Cottrell et al. [33]. In this context, although insulin and glucose play central roles in energy homeostasis, neither plasma insulin or glucose was different across any of the groups studied and thus other markers of energy metabolism need to be examined in future independent studies.

In the present study, the femoral cortical cross-sectional thickness was significantly greater in the WEX than in the CON group. Cross-sectional thickness may increase due to an increase in periosteal volume alone, a decrease in endosteal/medullary volume alone, or a combination of both. There were no significant differences between WEX and CON groups with respect to these two variables when separately analyzed, but when combined in the form of mean cortical thickness the small changes resulted in a significant difference in the latter morphological property of the WEX compared to the CON group. Despite the periosteal volume of the WEX group being almost identical to that of the CON group, when corrected to body weight the former was 6.7% greater than the latter; a similar but lesser difference was also present in endosteal volume. Although these changes were small, there appeared to be a biological effect on cortical bone mass and thickness, presumably associated with the different exercise regimen of the two groups. Because bending during locomotion is likely to be greater in the mid-diaphysis than at the site chosen for cortical bone scanning in this study, differences in cortical thickness and possibly other morphological features or properties may have been even more marked at the middiaphyseal site, and possibly in the BPS group also.

Cortical porosity is made up of resorption cavities, vascular, lacunar-canaliculi, and collagen-apatite porosities [34] and contributes to the mechanical properties of cortical bone [35]. Small changes in porosity can result in considerable loss of bone strength [36]. Percentage of closed porosity was significantly higher in the WEX than in the CON group. Related properties, percentage of open porosity, and total porosity tended to be lower in the WEX compared to the CON group, and these differences approached statistical significance.

We demonstrated differential effects in the two exercise groups as compared to the control group. We could not record all parameters of the two regimens, such as number of cycles, duration of exercise, rest insertion, and forces acting in the hindquarters of the animals. Thus standardisation of the two exercise regimens and comparison between them was not directly possible. Due to the short duration of exercise (17 days), as dictated by the interval between weaning and puberty, this experiment was designed to determine if high frequency activity (running) in young rats in a free-running wheel or a low frequency activity (lifting body weight in bipedal standing) compared to control rats would be associated with detectable effects on phenotype.

## 5. Conclusions

Collectively our observations indicate that the cortical bone microenvironment was affected by the exercise stimulus and responded accordingly. Fifteen days of moderate voluntary exercise was sufficient to affect muscle mass, localized adiposity, and bone microarchitecture in prepubertal male rats. Importantly, these changes were independent from any adverse stress responses or changes in caloric intake of the animals. Response to exercise was modality dependent with wheel exercise having a significant effect on the musculoskeletal system and localized adipose reserves. On the other hand, bipedal stance, a form of resistance training, did not significantly affect any of the parameters examined. The knowledge that early-life short-term voluntary exercise can affect three major components of body composition (muscle, bone, and fat) can be exploited to investigate its efficacy as an early-life intervention to improve health outcomes later in life, particularly in those at risk for early obesity.

## Figures and Tables

**Table 1:** Comparison of bone morphometric properties as determined by *µ*CT. Trabecular and cortical variables were measured at the distal physis of the right femur in the exercise groups. WEX rats had higher cortical cross-sectional thickness. There was a trend towards differences in open porosity and total porosity between CON and WEX but these did not reach statistical significance (*P* = 0.0610 and *P* = 0.0673, resp.). CON: control; BPS: bipedal stance; WEX: wheel exercise. Data are mean ± SEM; *n* = 8 per group. ^*^ *P* < 0.05 for CON versus WEX.

Trabecular bone	CON	BPS	WEX
Bone volume (mm^3^)	0.71 ± 0.06	0.66 ± 0.08	0.70 ± 0.06
Percent bone volume (%)	4.41 ± 0.29	4.30 ± 0.47	4.35 ± 0.38
Bone mineral density (g/cm^3^)	0.089 ± 0.006	0.093 ± 0.009	0.095 ± 0.006
Trabecular thickness (mm)	0.04 ± 0.00	0.04 ± 0.00	0.04 ± 0.00
Trabecular separation (mm)	0.50 ± 0.04	0.49 ± 0.04	0.51 ± 0.03
Trabecular number (mm^−1^)	1.15 ± 0.07	1.08 ± 0.12	1.08 ± 0.09
Trabecular pattern factor (mm^−1^)	28.28 ± 0.79	28.02 ± 1.01	27.04 ± 0.9
Structure model index (no units)	2.07 ± 0.03	2.1 ± 0.04	2.07 ± 0.04
Connectivity density (1/mm^3^)	82.07 ± 20.02	74.30 ± 26.27	73.85 ± 26.11

Cortical bone	CON	BPS	WEX

Periosteal volume (mm^3^)	7.49 ± 0.15	7.27 ± 0.16	7.47 ± 0.24
Endosteal volume (mm^3^)	5.51 ± 0.14	5.26 ± 0.14	5.40 ± 0.20
Bone volume (mm^3^)	1.98 ± 0.03	2.01 ± 0.04	2.07 ± 0.05
Percent bone volume (%)	26.48 ± 0.54	27.67 ± 0.50	27.81 ± 0.46
Tissue mineral density (g/cm^3^)	1.084 ± 0.019	1.129 ± 0.018	1.126 ± 0.009
Mean polar moment of inertia (mm^4^)	6.48 ± 0.22	6.35 ± 0.29	6.73 ± 0.37
Cross-sectional thickness (mm)	0.098 ± 0.005	0.112 ± 0.004	0.113 ± 0.004^*^
Open porosity (%)	12.31 ± 1.19	9.68 ± 0.77	9.40 ± 0.66
Total porosity (%)	12.60 ± 1.15	10.07 ± 0.75	9.83 ± 0.66
